# Structural and Dynamic Features of the Recognition of 8-oxoguanosine Paired with an 8-oxoG-clamp by Human 8-oxoguanine-DNA Glycosylase

**DOI:** 10.3390/cimb46050253

**Published:** 2024-04-29

**Authors:** Maria V. Lukina, Polina V. Zhdanova, Vladimir V. Koval

**Affiliations:** 1 Institute of Chemical Biology and Fundamental Medicine, Siberian Branch of Russian Academy of Sciences (ICBFM SB RAS), Novosibirsk 630090, Russia; p_chalova@niboch.nsc.ru; 2 Department of Natural Sciences, Novosibirsk State University, Novosibirsk 630090, Russia

**Keywords:** DNA damage recognition, 8-oxoguanosine, protein–DNA interaction, cytosine analog, MD simulations

## Abstract

8-oxoguanine (oxoG) is formed in DNA by the action of reactive oxygen species. As a highly mutagenic and the most common oxidative DNA lesion, it is an important marker of oxidative stress. Human 8-oxoguanine-DNA glycosylase (OGG1) is responsible for its prompt removal in human cells. OGG1 is a bifunctional DNA glycosylase with N-glycosylase and AP lyase activities. Aspects of the detailed mechanism underlying the recognition of 8-oxoguanine among numerous intact bases and its subsequent interaction with the enzyme’s active site amino acid residues are still debated. The main objective of our work was to determine the effect (structural and thermodynamic) of introducing an oxoG-clamp in model DNA substrates on the process of 8-oxoG excision by OGG1. Towards that end, we used DNA duplexes modeling OGG1-specific lesions: 8-oxoguanine or an apurinic/apyrimidinic site with either cytidine or the oxoG-clamp in the complementary strand opposite to the lesion. It was revealed that there was neither hydrolysis of the N-glycosidic bond at oxoG nor cleavage of the sugar–phosphate backbone during the reaction between OGG1 and oxoG-clamp-containing duplexes. Possible structural reasons for the absence of OGG1 enzymatic activity were studied via the stopped-flow kinetic approach and molecular dynamics simulations. The base opposite the damage was found to have a critical effect on the formation of the enzyme–substrate complex and the initiation of DNA cleavage. The oxoG-clamp residue prevented the eversion of the oxoG base into the OGG1 active site pocket and impeded the correct convergence of the apurinic/apyrimidinic site of DNA and the attacking nucleophilic group of the enzyme. An obtained three-dimensional model of the OGG1 complex with DNA containing the oxoG-clamp, together with kinetic data, allowed us to clarify the role of the contact of amino acid residues with DNA in the formation of (and rearrangements in) the enzyme–substrate complex.

## 1. Introduction

**Figure 1:**
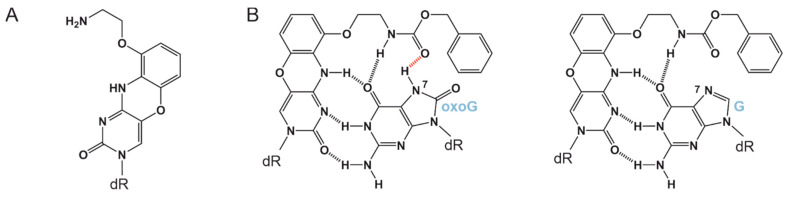
(**A**). G-clamp structure and (**B**). the difference in hydrogen bond formation between the oxoG-clamp and either oxidized or normal guanosine [22].

After the very first studies showing the potential of antisense oligonucleotides to alter a protein’s expression level [1,2] by various mechanisms [3,4,5], the rapid development of approaches for obtaining modified oligonucleotides that would have both higher resistance to nonspecific nucleases and a stronger affinity for a target DNA site began [6,7]. One way to obtain such oligonucleotides is nucleobase modification [8]. A phenoxazine derivative named G-clamp is a tricyclic heterocycle that has been proposed as a cytosine analog [9] (Figure 1A). It can form four hydrogen bonds with guanine and increases DNA duplex stability [9,10,11], thus enhancing the affinity and selectivity of target DNA binding [12]. G-clamp-containing oligonucleotides are actively improving [13,14] and quite useful for various biomedical and research applications [15,16,17,18,19,20]. The oxoG-clamp has emerged as an extension of the G-clamp idea [21]. This is a modified G-clamp, which has an additional tail, which is capable of forming an additional hydrogen bond with the hydrogen of the N7 atom, which is absent in normal guanine but present in 8-oxoguanine [22]: a hydroxylated guanine derivative, which, in aqueous solutions, exists mainly in the keto form [23,24] and differs from guanine by two additional atoms in moieties H–N7 and O–C8 (Figure 1B). The oxoG-clamp can bind to normal guanine, thereby forming four hydrogen bonds, or to oxidized guanine, thus forming one more additional hydrogen bond (five in total), but especially interesting and useful is the fact that this derivative has different fluorescent properties depending on the opposite base. OxoG-clamp fluorescence is quenched efficiently by coupling with oxidized guanine [25,26].

8-oxoguanine (oxoG) is a result of the action of exogenous or endogenous reactive oxygen species on DNA [27,28] and is one of the most common oxidative lesions [29]. OxoG has strong mutagenic properties. It is able to form Hoogsteen base pairs with a nucleobase without significant distortion of the B-DNA helix and causes the misincorporation of adenine during replication, since DNA polymerases bypass oxoG with different accuracies [30,31,32].

Furthermore, the question of 8-oxoguanine detection and estimation of its levels in DNA, cells, and tissues is of great scientific and diagnostic interest, because there is ample evidence that oxoguanine levels are elevated during carcinogenesis and other pathological processes [33,34,35]. Various methods have been proposed for 8-oxoguanine detection, but they all have their limitations and drawbacks [36,37,38,39,40,41]. Therefore, a specific fluorophore able to selectively bind to 8-oxoguanine and distinguish it from numerous intact bases and nucleotides may be a useful practical tool for 8-oxoguanine quantification in or outside the DNA. Normally, 8-oxoguanine in human cells is promptly removed via the base excision repair pathway [42,43] by 8-oxoguanine-DNA glycosylase (OGG1), which is the key DNA repair enzyme [44,45]. OGG1 is a bifunctional DNA glycosylase and acts on DNA like both N-glycosylase and AP lyase [46,47].

8-oxoG recognition in the OGG1 active site is mediated by the formation of specific contacts between the damaged base and several amino acid residues of the enzyme. OGG1 recognizes the ureic moiety of 8-oxoG, including the C8-carbonyl group and atoms N7 and N9. The N7 atom is engaged in a hydrogen bond with the carbonyl group of the Gly42 residue [48]. Among all the active site contacts of 8-oxoG, this is the only interaction with the Gly42 residue that intact guanine does not have. It is reasonable to hypothesize that this hydrogen bond is responsible for discriminating 8-oxoG from G. It should be noted that the critical amino acid Gly42 is placed into the β-fold domain located in the hOGG1–DNA contact region.

In addition to the protein–DNA contact mentioned above, other amino acid residues of the OGG1 active site are also involved in oxoG recognition. Residues Phe319 and Cys253 interact with the π-system of 8-oxoG on two opposite sides, thereby forming a sandwich structure. After damaged-base eversion, the cytosine opposite the oxoG is coordinated by hydrogen-bonding interactions with Arg154, Arg204, and Asn149 [47].

In this study, we wanted to assess the properties of short DNA duplexes containing the oxoG–oxoG-clamp pair in order to evaluate introduction of the oxoG-clamp into a DNA structure.

We were interested in studying the particular features of OGG1 enzyme interaction with oxoG_clamp-containing DNA duplexes. Additionally, we wanted to take advantage of the unique fluorescent properties of the oxoG-clamp. Based on the idea of increasing fluorescent signal as an indication of 8-oxoguanine excision [49], in our work we decided to insert the oxoG_clamp opposite to the damage to investigate the initial steps of the recognition of oxoguanine and its eversion from the DNA duplex by OGG1.

## 2. Materials and Methods

### 2.1. The Enzyme and Oligodeoxynucleotides (ODNs)

All ODNs were synthesized in the Laboratory of Synthetic Biology at the ICBFM SB RAS by the phosphoramidite method on an ASM-800 synthesizer (Biosset Ltd., Novosibirsk, Russia) using Glen Research monomers (Glen Research, part of Maravai LifeSciences, Sterling, VA, USA). Carboxyfluorescein (FAM) was also introduced as a corresponding phosphoramidite during ODN synthesis. The purity of all ODNs was verified by denaturing electrophoresis in a 20% polyacrylamide gel.

When needed, the ODNs were ^32^P-labeled using polynucleotide kinase T4 (SibEnzyme, Novosibirsk, Russia). Namely, 30 pmol of [γ-^32^P]ATP and 5 µM ODN were incubated for 30 min with 10–20 U of phage T4 polynucleotide kinase at 37 °C in a reaction buffer (SibEnzyme, Novosibirsk, Russia).

An apurinic/apyrimidinic (AP) site containing ODN was prepared by the incubation of 0.1 μmol of an ODN containing dU at the position of the prospective AP site for 14 h at 37 °C with 15 U of uracil-DNA glycosylase (SibEnzyme, Novosibirsk, Russia) in 150 µL of reaction buffer (SibEnzyme, Novosibirsk, Russia). The reaction product was purified by reverse-phase HPLC on a Nucleosil 100-5 C18 column (Macherey-Nagel GmbH, Dueren, Germany); the fraction containing the product was concentrated and then converted into lithium salt by means of a Sep-Pak Plus C18 cartridge (Waters, Milford, MA, USA) [50].

The human *OGG1* (isoform 1a) gene was cloned into the pET-15b plasmid and expressed via induction in *Escherichia coli* BL21(DE3), as described in detail previously [51]. The purity of the enzyme was checked by SDS-PAGE, with Coomassie Blue staining.

### 2.2. Stopped-Flow Measurement

Kinetic traces were recorded using an SX.18MV stopped-flow spectrophotometer (Applied Photophysics, Leatherhead, Surrey, UK). The fluorescence excitation wavelength was 365 nm, which corresponds to the absorption maximum of the oxoG-clamp fluorophore. All the experiments were conducted under the same conditions as the experiments on the electrophoretic separation of reaction products in a polyacrylamide gel: 25 °C and a buffer consisting of 50 mM Tris-HCl (pH 7.5), 50 mM KCl, 1 mM EDTA, 1 mM DTT, and 9% of glycerol. In each experiment, the concentration of the ODN duplex was constant at 10^−6^ M, and the concentration of the enzyme was varied in the micromolar range. Changes in fluorescence intensity of the oxoG-clamp were observed at wavelengths above 420 nm using a GG-420 light filter (Schott, Mainz, Germany). The dead time of the instrument was 1.4 ms. Each kinetic curve was a result of averaging at least four individual experimental curves.

### 2.3. Kinetic Data Processing

The kinetic parameters were calculated by global nonlinear fitting in the DynaFit software v. 4 (BioKin, Watertown, MA, USA) [52] from the temporal behavior of the fluorescence curves obtained at different enzyme concentrations. This software numerically integrates systems of ordinary differential equations corresponding to proposed kinetic schemes and performs nonlinear least-squares regression analysis. The overall shape of a fluorescent curve gives preliminary information about the possible number of elementary steps in the overall mechanism. To determine the minimal kinetic scheme satisfactorily describing our set of experimental curves, global fitting was started with the kinetic curves’ initial part, which could be described as a single-exponential stage. Further data processing was carried out by step-by-step expansion of the experimental curves’ time range subjected to data processing and by the addition of more steps to the mechanism. Our approach has been described in more detail in previous publications [50,53,54].

### 2.4. An Assay of Cleavage by OGG1

To analyze the products resulting from DNA substrates after the interaction with OGG1, the reaction was conducted under the same conditions as the stopped-flow experiments, except for the fact that the substrate ODNs were ^32^P-labeled. The reaction was terminated at desired time points by adding a loading dye solution containing 8 M urea. To determine the rate of nicked-product formation (AP lyase enzymatic activity), samples were directly analyzed by the separation of a reaction mixture in a denaturing 20% polyacrylamide gel. To analyze the rate of AP site formation from the substrate containing oxoG (the N-glycosylase activity), before the PAGE procedure, the samples were incubated in 0.3 M NaOH at 56 °C for 20 min, then HCl was added to bring the solutions to a pH of 7. The gels were exposed to Agfa CP-BU X-ray film (Agfa-Geavert, Mortsel, Belgium). In the experiments with FAM-labeled duplexes, imaging was performed on an Typhoon RGB Scanner (Cytiva, Wilmington, DE, USA). The degree of substrate cleavage was determined using the Gel-Pro Analyzer software v. 3.2 package (Media Cybernetics, Rockville, MD, USA).

### 2.5. Determination of the Melting Temperature of DNA Duplexes

Experiments on thermal denaturation of duplexes were conducted on a Varian Carry 300-Bio spectrometer [described in detail in Ref. [55]]. For the DNA solutions (2 µM of each strand) in a reaction buffer (50 mM Tris-HCl, pH 7, 5 mM, 50 mM KCl, 1 mM EDTA, 1 mM DTT, 9% glycerol) and for the empty reaction buffer (control), optical absorption curves were recorded at 260, 270, and 330 nm during heating from 5 to 95 °C at 0.5 °C/min increments (in a quartz cuvette, 0.2 cm optical path length, Peltier thermostated multicell holder [6 × 6]). The optical density curves obtained at wavelengths 260 and 270 nm were employed to calculate the melting temperature of the DNA duplexes; the data obtained at wavelengths of 330 nm were used to assess the correctness of the experiment and correct the baseline [56]. After mathematical processing—smoothing and differentiation (in OriginPro 2021)—the maxima in the differential melting curves were designated as the estimated melting temperatures for each DNA duplex.

### 2.6. MD Simulations

We set up models of complexes of wild-type hOgg1 with 13-mer ODN duplexes containing oxoG and either C or oxoG-clamp at the 7th position of one strand. The crystal structure of the hOgg1 complex with an ODN duplex (Protein Data Bank [PDB] ID: 1EBM) served as the starting structure [47]. In the starting structure, Gln at position 249 was replaced by Lys according to the wild-type amino acid sequence. We modified the ODN sequences using Chimera 1.16 [57]. Force field parameters for the oxoG-clamp residue were obtained with the help of a software package [58] when parameterized using basis set 6-31G* [59]. The procedure yielded a set of OGG1 complexes with different DNA duplexes: oxoG/oxoG-clamp, oxoG/C where oxoG was inside the pocket (active center), and oxoG/C where oxoG was in the duplex. We used the obtained models to perform molecular dynamics (MD) simulations.

MD simulations were performed in the Amber20 software [60], with an accelerated GPU code [61,62]. Force field ff14SB [63] was applied to the protein and force field bsc1 [64] to the DNA. To eliminate undesirable contacts, we started the MD simulations by minimizing (500,000 minimization steps) the energy of the complexes in an implicit solvent model. Then, a water environment (the TIP3P water model with 8 Å cubic periodic conditions) was added to the optimized models of the complexes. The amount of water in the simulated cell ranged from 12,199 to 12,777 molecules. Sodium ions were utilized to neutralize the negative charge in the periodic cell. Minimization of the systems via the explicit solvent model was carried out in two steps. In the first step, the solvent molecules were relaxed while the complex remained fixed, and, during the second step, the whole system was minimized. The fixed complex system was heated for 125 ps from 1 to 300 K using pmemd.cuda. The system’s density was equilibrated for 50 ps at a constant pressure of 1 bar, followed by equilibration at a constant pressure of 1 bar and 300 K for 500 ps. The MD simulations were performed for 100 ns in an NPT ensemble (1 bar, 300 K). The obtained trajectories were analyzed by means of Chimera 1.16 and cpptraj [65].

## 3. Results and Discussion

**Figure 2:**
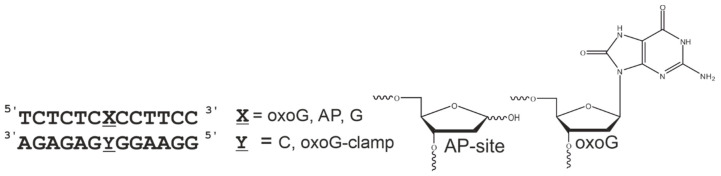
Our model DNA duplexes.

In our work, we used DNA duplexes containing (at a central position) guanosine or a specific lesion (8-oxoguanosine or AP site), which are recognized and can be processed by human 8-oxoguanine-DNA glycosylase. Either normal cytosine or the oxoG-clamp was located opposite to the lesion (Figure 2). This approach allowed us to evaluate the influence of the oxoG-clamp on the structure of the duplexes and on the parameters of the interaction with the enzyme.

### 3.1. Properties of DNA Duplexes Containing the oxoG_clamp

**Figure 3:**
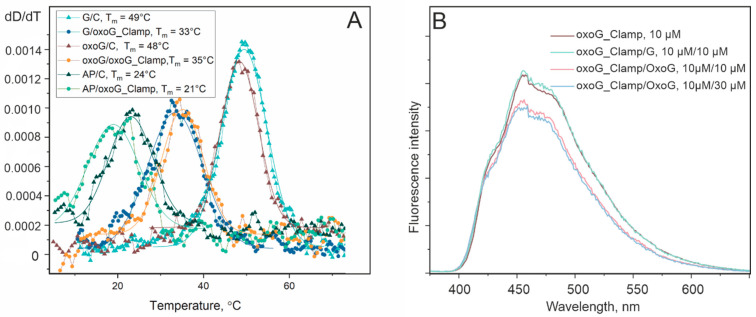
(**A**). Melting curves of DNA duplexes. (**B**). Fluorescence changes (in a.u.) after addition to single-stranded ODN containing the oxoG-clamp (brown trace) of a complementary strand containing guanine (green trace) or oxoguanine (blue and pink traces) opposite to the oxoG-clamp.

To evaluate the effect of the oxoG-clamp on the stability of the DNA duplexes, melting curves of 2 μM solutions of duplexes in a reaction OGG1 buffer were recorded. All the melting curves had a sigmoidal shape typical for the thermal denaturation of DNA duplexes. The melting temperatures were estimated as the maximum in a differential bell-shaped melting curve (Figure 3A). One pioneering work on the G-clamp revealed an increase in the melting temperature for the 11-mer DNA duplexes containing a single G-clamp [10]. In principle, tricyclic cytosine analogs and phenoxazine derivatives in particular have been proposed to enhance binding and helix stability in DNA and hybrid RNA/DNA duplexes, both by increasing the number of hydrogen bonds (on both sides of the Watson–Crick and Hoogsteen faces) and by enhancing stacking interactions with neighboring nucleotides, compared to the standard Watson–Crick G–C pair [9]. In ref. [26], no significant difference in the melting temperatures was detected when the oxoG-clamp was introduced into the ODN duplexes. Under our conditions, it was found that the replacement of C-opposite oxoG with the oxoG-clamp reduced the melting temperature of the duplex by 13 °C (48 vs. 35 °C). These data indicate a significant destabilization of the duplex by this substitution. Despite the possibility of the formation of five hydrogen bonds, this bulky compound in place of the nucleobase had no stabilizing effect on the DNA duplex in our case. Apparently, the steric effect and the inability of the bases adjacent to the damage to engage in stacking interactions owing to spatial perturbations caused more serious disturbances. The discrepancy between our data and the effect observed earlier for G/G_clamp-containing duplexes may be explained both by the presence of extra oxygen atom at the guanine C8 atom and by the emergence of the bulky “tail”, i.e., a benzyloxycarbonyl group (Cbz, benzyloxycarbonyl), which prevents the proper coordination of neighboring nucleobases for a stacking interaction. The examination of the results of the thermal denaturation of the duplexes containing the G–C or G–oxoG-clamp pair in the middle also indicated destabilization of the duplex when the oxoG-clamp was present in it, with the effect being even more pronounced: ΔT = 16 °C (melting temperature decreased from 49 to 33 °C when cytosine was replaced with the oxoG-clamp). In the experiments with the AP site–containing duplex, there was almost no change in the melting temperatures (24 and 21 °C for the C- and oxoG-clamp-containing duplexes, respectively). This finding also confirms the hypothesis of spatial hindrances introduced by the bulky analog of the nucleobase. In the case of AP site–containing duplexes, the additional space opposite to the oxoG-clamp owing to the absence of the nucleobase provides opportunities for the proper packing of the oxoG-clamp within the ODN structure.

In an assay of the fluorescent properties, it was shown that the addition of an equimolar amount of the strand containing 8-oxoguanosine to a 10 μM solution of an oxoG-clamp-containing strand decreased the fluorescent signal from the oxoG-clamp by approximately 20% (Figure 3B). At the same time, the addition of an excess of the oxoG-containing strand did not lead to a further decrease in fluorescence. The observed quenching of the fluorescence was less than that reported in ref. [21], where experiments with the titration of free nucleosides (containing protective TBDMS groups on the 3′-O and 5-O′ atoms) were conducted. This discrepancy can be explained by the fact that the oxoG-clamp in the ODN is subject to additional steric restrictions on the maximum proximity to oxoG; this proximity is required for effective fluorescence quenching. Moreover, the magnitude of fluorescence quenching seems to be significantly affected by buffer composition (in particular, by the ionic strength of the solution and the presence of Mg^2+^ ions). This observation may explain a discrepancy between our results and the experimental findings in ref. [26], where the oxoG-clamp incorporated into a 16-mer monopurine nucleotide was found to have 25% of the initial fluorescence after a complementary strand with oxoG had been added (up to 80% of the initial fluorescence when a G-containing complementary strand was added). Under our conditions, the addition of the strand containing normal guanosine to the initial solution (of the oxoG-clamp-containing strand) did not cause a decrease in oxoG-clamp fluorescence (Figure 3B). The selective quenching of oxoG-clamp fluorescence upon binding to oxoG makes our system and buffering conditions convenient for detecting the state of 8-oxoguanine within the duplex: whether it interacts with the base analog opposite the damage or is displaced from the DNA strand.

### 3.2. Kinetics of OGG1 Interaction with DNA Duplexes Containing the oxoG-clamp

**Figure 4:**
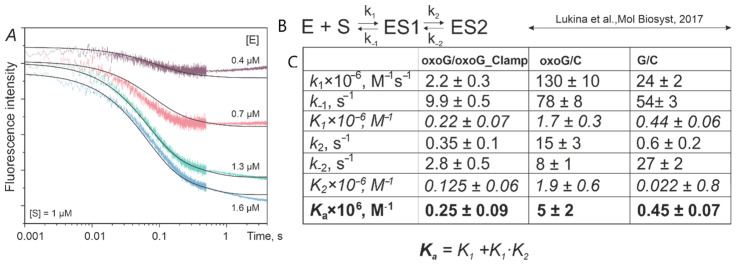
Kinetic fluorescent traces (in a.u, panel (**A**)), the mechanism (**B**), and rate constants (**C**) for the interaction of 1 μM OGG1 with ODN duplex oxoG/oxoG-clamp (0.4–1.6 μM concentration range). Kinetic parameters for oxoG/C and G/C duplexes are from [54].

Our hypothesis was that the specific quenching of oxoG-clamp fluorescence by 8-oxoguanine would allow us to register the key moment of the damaged-base eversion into the OGG1 active site. Thus, the removal of the damaged base from the stacking and DNA helix structure should have released the oxoG-clamp and enhanced the fluorescent signal. Oxoguanine eversion is one of the initial and pivotal steps of the enzymatic process, occurring in the millisecond range. Therefore, we chose the kinetic stopped-flow technique, which allowed us to quickly mix the enzyme solution with the substrate solution and record changes in the fluorescent signal from the millisecond time range. All the experiments were carried out under single-turnover conditions. We expected to see an oxoG-clamp fluorescence increase caused by the breakage of bonds with 8-oxoguanine as it was flipped out into a base-binding pocket. By contrast, only a slight decrease in the fluorescent signal’s intensity at time points up to 1 s was recorded (Figure 4A). These curves were well consistent with the kinetic mechanism involving two reversible stages (Figure 4B). When processing the concentration series of curves in the Dynafit 4.0 software, the two-step kinetic mechanism was selected, and the rate constants of the elementary stages of enzyme–substrate binding were determined. It should be noted that enzyme–substrate complex formation was additionally confirmed using an EMSA analysis (Figure S1). A comparison of the kinetic parameters with the data that we obtained earlier for the enzyme with duplexes containing C in the complementary strand (Figure 4C) [54] revealed that the reaction between OGG1 and ODN duplex oxoG/oxoG-clamp was more similar to the features of the interaction of hOgg1 with nonspecific duplex G/C. The K_a_ values (overall binding constants) for ODN duplexes oxoG/oxoG-clamp and G/C were (0.25 ± 0.09) × 10^6^ and (0.45 ± 0.07) × 10^6^ M^−1^, respectively. On the other hand, the constant of the binding of OGG1 to ODN duplex oxoG/C was (5 ± 2) × 10^6^ M^−1^, at least an order of magnitude higher. The ratios of the rate constants of the forward and reverse elementary steps (Figure 4C, K_1_ and K_2_) were also consistent when duplexes oxoG/oxoG-clamp and G/C were compared and were 1–2 orders of magnitude lower than those for the preferable substrate (ODN duplex oxoG/C). Thus, there were no signs of 8-oxoguanine eversion from the DNA strand either in terms of the nature of the signal changes or in the kinetic parameters. A slight decrease in the intensity of oxoG-clamp’s fluorescent signal can be explained either by an alteration in the fluorophore’s microenvironment (a change in the hydrophobicity and polarity of the medium, which may affect the quantum yield of the fluorescence) or, unpredictably, by the additional convergence of 8-oxoguanine and phenoxazine in the duplex during protein–DNA binding, improving the efficiency of oxoG-clamp fluorescence quenching.

**Figure 5:**
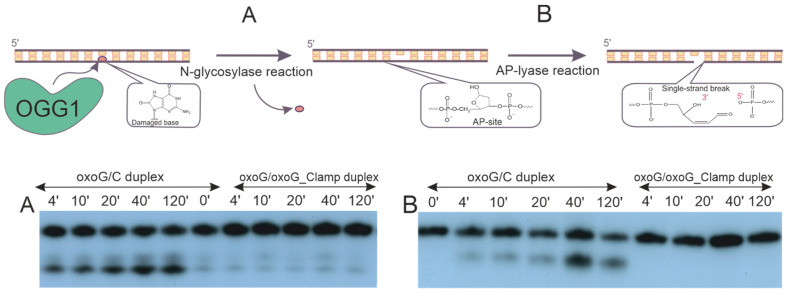
General scheme of DNA cleavage catalyzed by OGG1 and the product accumulation rates for the N-glycosylase (**A**) and AP lyase reactions (**B**) of OGG1 with ODN duplex oxoG/C or oxoG/oxoG-clamp.

The product formation rates for duplexes oxoG/C and oxoG/oxoG-clamp mixed with OGG1—also under single-turnover conditions—were analyzed by PAGE. Since the OGG1 has two enzymatic activities, we needed to record two reaction products (Figure 5). The nicked product formed after both glycosylase and lyase reactions was directly observed by the movement of the short product in the gel (Figure 5B). To visualize the amount of product containing the AP site after the first N-glycosylase step, the oligonucleotide was treated by adding NaOH to the reaction mixture in order to chemically cleave the AP site before PAGE.

In the case of ODN duplex oxoG/oxoG-clamp, no formation of either N-glycosylase or AP lyase reaction products was recorded throughout the examined period (2 h) (Figure 5). By contrast, under equivalent conditions, ~50–60% of oxoG was excised from duplex oxoG/C by the enzyme (Figure 5A), and ~40–45% of the AP site–containing product underwent subsequent cleavage, with the formation of a β-elimination product (Figure 5B).

The extent of substrate cleavage by OGG1 was also determined for the DNA duplex devoid of the base opposite the oxoG-clamp. An ODN duplex containing the AP–C pair has also been previously recognized by hOGG1 and has undergone a β-elimination reaction [47,50,66], whereas, in our work, the incubation of hOGG1 with ODN duplex AP/oxoG-clamp for 3 h did not lead to nicked-product formation (data available on demand). Moreover, an attempt to accelerate the enzymatic activity via the introduction of 500 μM 8-bromoguanine (an analog of 8-oxoguanine) into the reaction mixture also failed to ensure product formation. By contrast, OGG1 β-elimination reaction acceleration (by at least an order of magnitude) after the addition of aminoguanine or bromoguanine to the enzyme–substrate mixture has been documented previously, not only for the wild-type enzyme [51,67,68] but also for its mutants [50]. The exact mechanism of this process is still debated. There is evidence supporting the “product-assisted catalysis” mechanism [67,68] and the mechanism of allosteric activation of the enzyme active site [69]. In the case of the oxoG-clamp opposite the AP site, the presence of bromoguanine does not facilitate the formation of a Schiff base as an intermediate, whose subsequent hydrolysis leads to the breakdown of the sugar–phosphate backbone [67,70]. Therefore, the inability to evert the damaged base into the enzyme’s active site pocket is not the only factor that prevents catalysis. Our reproduction of conditions simulating “deeper” stages of the enzymatic process (pre-β-elimination complex) also did not lead to the appearance of reaction products when the oxoG-clamp was present in the undamaged strand. Substrate specificity has already been demonstrated for the OGG1 enzyme in terms of the base opposite the lesion [66,68]. These data allow us to suggest that incorrectly formed DNA–protein contacts with the base opposite to the damage play a critical role in the loss of enzyme activity. To identify the structural reasons behind why OGG1 completely loses the ability to both recognize the oxidized base and cut the sugar–phosphate backbone when facing the oxoG-clamp, our subsequent analysis comprised molecular modeling.

### 3.3. MD Simulations

The results of MD modeling provided information on the stability of the free protein and its complexes as well as their dynamic behavior. We started our computational simulations of the structures of interest by obtaining a three-dimensional (3D) structure of hOgg1 complexes with 13-mer DNA duplexes containing oxoG at the 7th position of one strand and either C or the oxoG-clamp opposite this base on the other strand. To determine the starting coordinates of all the atoms, we utilized the crystal structure of human 8-oxoguanine-DNA-glycosylase (OGG1) bound to a substrate oligonucleotide (PDB ID: 1EBM). Nucleic acid sequences were modified using UCSF Chimera 1.16. The initial models consisted of 314 amino acid residues and a 13-mer DNA duplex. It should be noted that the nucleotide sequences in the simulated structure of the complex matched the sequences used in the experiments. To assess the reproducibility of the results for each model, we performed two MD simulations with different random initial values.

**Figure 6:**
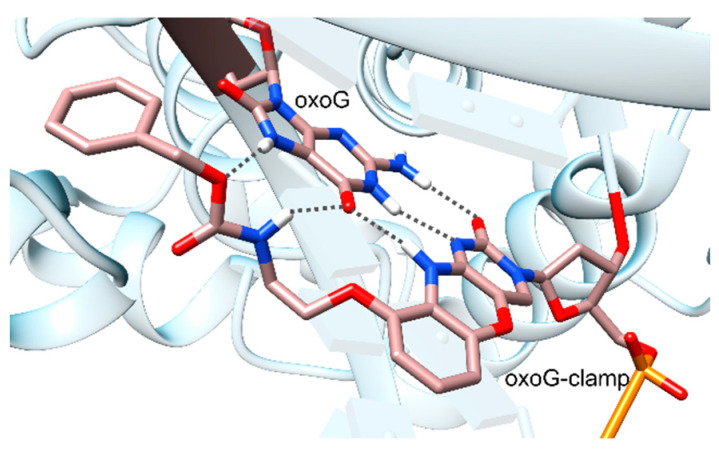
Hydrogen bonds formed by oxoG and the oxoG-clamp.

The analysis of the MD trajectories for the OGG1 complex with the DNA duplex containing the oxoG–oxoG-clamp pair indicated the stability of both the protein and DNA components of the complex throughout the entire MD trajectories. For instance, the root mean square deviations (RMSDs) for the protein during 100 ns ranged between 2 and 3 Å. Figure 6 shows the hydrogen bonds formed by oxoG and the oxoG-clamp, which remained stable over 100 ns according to the RMSD.

**Figure 7:**
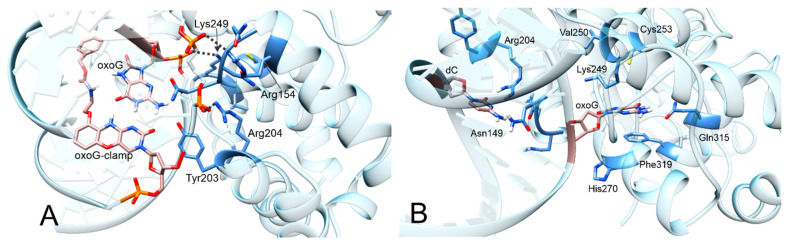
(**A**). The complex of oxoG with the oxoG-clamp surrounded by amino acid residues, while the active center of the enzyme remains unoccupied. (**B**). oxoG surrounded by amino acid residues of the enzyme’s active center.

**Figure 8:**
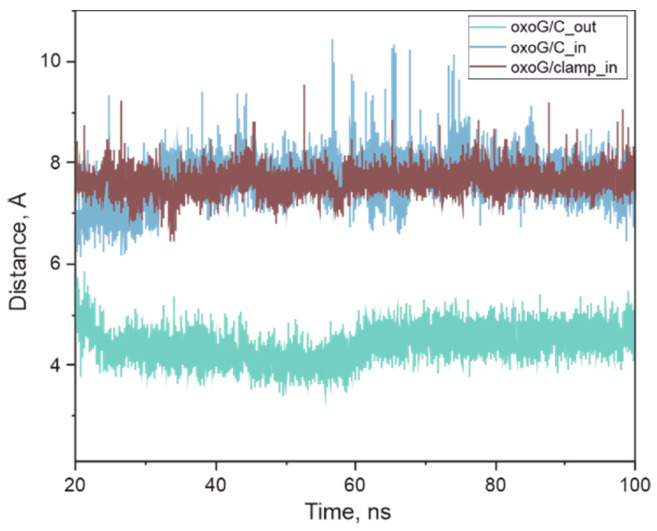
Distances between the N atom of the Lys249 side chain and the C1’ atom of oxoG in OGG1 complexes with DNA duplexes containing the oxoG-clamp opposite the damage (brown trace) and a duplex with C opposite the damage where oxoG is stacked (blue trace) or flipped out onto the enzyme active site (green trace).

Because oxoG was paired with the oxoG-clamp, the active center of the enzyme remained unoccupied (Figure 7A). Initially, amino acid residues Asn149, Arg154, Arg204, and Tyr203 coordinated the oxoG-clamp, but, over time, the duplex became slightly distant, and the oxoG-clamp was no longer coordinated. Of note, the phenyl ring of the oxoG-clamp was supposed to participate in the stacking interaction, but, due to a rather long linker, it appeared to be mobile and did not engage in the interaction (despite the initial positioning). Because the interaction of Lys249 with oxoG is known to be important, we analyzed the distance between the N atom of the Lys side chain and C1’ in oxoG. The distance between these atoms remained ~8 Å (Figure 8). Thus, the MD simulation showed that, for the complex in question, the enzymatic process was not possible due to the retention of the oxoG–oxoG-clamp pair.

We examined the models of complexes in which oxoG was paired with C in the opposite strand of the duplex and those in which the DNA substrate was in the reaction-ready state, with oxoG positioned in the active center of OGG1.

In the model where oxoG was interacting with cytosine, the protein had a slightly higher stability, with the RMSDs mainly fluctuating around 2 Å. Of note, the oxoG–C pair within the duplex was unstable, and oxoG slightly protruded (was everted) from the duplex over time, although it did not enter the active center of the enzyme. The distance between the N atom of Lys249 and the C1’ atom of oxoG remained approximately 8 Å throughout the MD simulation, similarly to the ODN complex oxoG/oxoG-clamp (Figure 8).

In the complex where oxoG was in the active center, the average distance between the N atom of Lys249 and C1’ atom of oxoG was 4.5 Å (Figure 8 and Figure 7B). While amino acid residue Phe319 was stacked with oxoG, Asn151 formed a hydrogen bond with the oxygen of the phosphate group located between oxoG and (−1)C (cytosine at position −1). Lys249 formed a hydrogen bond with an oxygen atom in the phosphate group located between oxoG and (+1)C (cytosine at position +1).

Residues Arg154 and Arg204 coordinated C opposite to oxoG: the nitrogen atoms (amino and imino groups) engaged in hydrogen bonds with the N3 atom and the oxygen atom of cytosine. The oxygen atom of Asn149 formed a hydrogen bond with the amino group of C. Asn150 participated in coordinating Arg154 in this study.

The structures of heterocyclic bases 8-oxoG and G differ only in two positions: the C8 atom is connected with either an O or H atom, and N7 has either an H atom or an unshared pair of electrons, respectively. Consequently, the H atom at the N7 position in oxoG can form a hydrogen bond with the carbonyl group of the Gly42 residue in the main chain, whereas G does not have this ability. The oxoG-clamp residue interacts with the carbonyl oxygen atom at position 8 of 8-oxoG, thereby preventing the oxoG base from protruding into the active center pocket of the hOgg1 enzyme.

## 4. Conclusions

In the presented work, we have shown that the oxoG_clamp fluorophore, which was initially proposed for the detection of oxidized purines in the DNA, seriously impedes the removal of 8-oxoG by the OGG1 enzyme.

We found that OGG1 could neither hydrolyze the N-glycosidic bond in oxoguanine nor cut the sugar-phosphate backbone of the oxoG-clamp–containing DNA duplex. This result is especially interesting when taken together with our finding that the presence of the oxoG-clamp itself in the complementary strand destabilizes the duplex. Our results once again highlight the critical importance of the contacts of active site amino acid residues not only with the damaged base but also with the opposite base. The amino acid residues responsible for discriminating between the oxoG and G participates in the binding to the oxidized base and opposite base in the recognition pocket include Gly42, Tyr203, Asn149, Arg154, Arg204, Phe319, and Asn151. The base opposite the damage has a major impact on the initial steps of the assembly of the enzyme–substrate complex. The presence of the oxoG-clamp residue prevents the oxoG base from protruding into the active center pocket of OGG1 and also hinders AP site cleavage, even if the oxidized base has already been removed. By combining the resulting 3D model of the hOGG1 complex with DNA and the oxoG-clamp along with kinetic data, we were able to gain insights into the role of specific amino acid–DNA contacts in the formation of (and rearrangements in) the enzyme–substrate complex.

The oxoG-clamp tag fluorescent properties can be convenient and usable for fluorescent detection of oxoguanosine in the DNA. However, when studying the features of protein–nucleic acid interactions, it is necessary to be particularly careful and pay attention during the design of the study to the perturbations that the oxoG-clamp introduces into the structure of the DNA and, most notably, to its influence on the functioning of DNA-binding enzymes.

It was demonstrated that DNA duplexes (under conditions optimal for hOGG1 repair enzyme operation) showed reduced stability when an oxoG-clamp was introduced instead of G, even though the oxoG-clamp molecule was initially considered to increase the stability of the DNA duplex (by both forming a larger number of hydrogen bonds and increasing the overlap for stacking interactions). This once again highlights the importance of how carefully oligonucleotides must be designed, for example, for antisense therapy, fluorescence imaging experiments, and other scientific and medical tasks.

OxoG-clamp presence in the opposite strand can not only affect the thermodynamic and structural characteristics of the oligonucleotide but also dramatically change the features of enzyme–substrate complex formation and the processing of repair enzymes and other DNA-binding proteins.

## Data Availability

Raw data are available upon request.

## Supplementary Materials

The following supporting information can be downloaded at https://www.mdpi.com/article/10.3390/cimb46050253/s1: Figure S1: Results of the EMSA experiments (native gel electrophoresis).
