# Supplementary material for: Structural and Dynamic Features of the Recognition of 8-oxoguanosine Paired with an 8-oxoG-clamp by Human 8-oxoguanine-DNA Glycosylase
Source: Curr Issues Mol Biol. 2024 Apr 29;46(5):4119–32. doi: 10.3390/cimb46050253 (PMC11120029; doi:10.3390/cimb46050253)

Supplementary Materials

Supplementary Figure S1. Results of the EMSA experiments (native gel electrophoresis).

A. DNA duplexes visualization by staining with SYBR Green

B. Visualization by FAM-label in DNA strain

The conditions of experiment are indicated in pictures

A

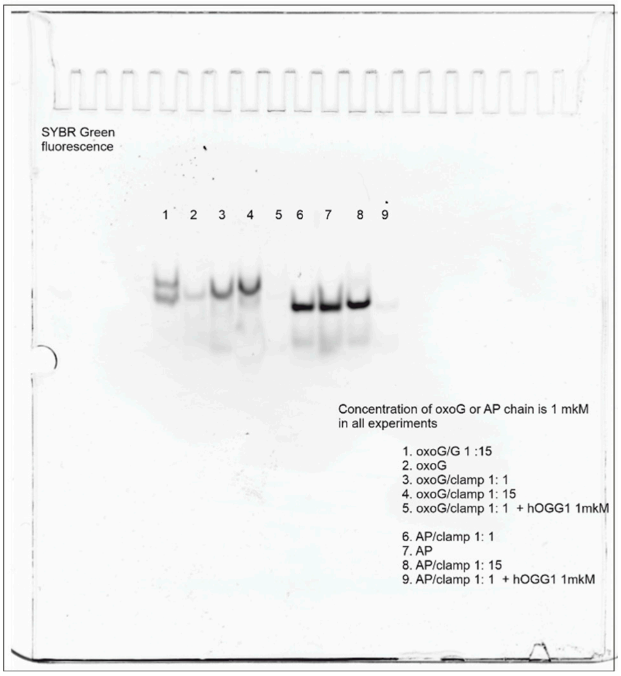

B

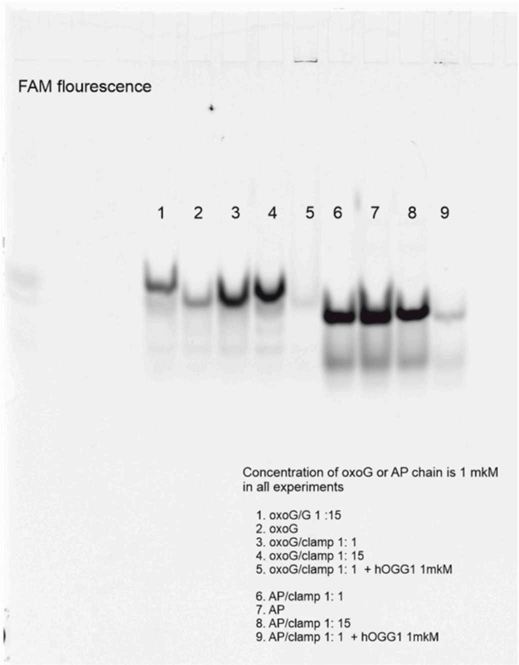

Supplement: Supplementary file 1 [file cimb-46-00253-s001.zip › cimb-2865765-supplementary.pdf]
